# Gamma-secretase inhibition combined with platinum compounds enhances cell death in a large subset of colorectal cancer cells

**DOI:** 10.1186/1478-811X-6-8

**Published:** 2008-10-24

**Authors:** Tamara Aleksic, Stephan M Feller

**Affiliations:** 1 Cell Signalling Group, Oxford University Department of Medical Oncology, Weatherall Institute of Molecular Medicine, Oxford, OX3 9DS, UK

## Abstract

**Background:**

Notch signalling is essential for the development and maintenance of the colonic epithelium. Its inhibition induces a differentiation phenotype in vivo and reduces adenomas in APC^min ^mice. Whether Notch signals are also required in colorectal cancer (CRC) has remained elusive. Therefore, 64 CRC cell lines were analysed for the occurrence of proteolytically processed, active Notch.

**Results:**

63 CRC lines contained a fragment with approximately the size of the Notch1 intracellular domain (NICD), which is required for signalling. Subsequent analyses with an antibody that specifically recognises the free Val1744 residue generated by γ-secretase-mediated cleavage of Notch1 showed that a subset of CRC cells lacks this specific Val1744-NICD. Surprisingly, inhibition of Val1744-NICD signalling with different γ-secretase inhibitors (GSI) did not lead to substantial effects on CRC cell line growth or survival. However, transient activation of Erk upon GSI treatment was detected. Since cisplatin relies on Erk activation for bioactivity in some cells, platinum compounds were tested together with GSI and enhanced cell killing in a subset of Val1744-NICD-positive CRC cell lines was detected. Erk inhibition ablated this combination effect.

**Conclusion:**

We conclude that γ-secretase inhibition results in activation of the MAP kinases Erk1/2 and, when used in conjunction, enhances cell death induced by platinum compounds in a large subset of colorectal cancer cell lines.

Furthermore the activation of Erk appears to be of particular importance in mediating the enhanced effect seen, as its inhibition abrogates the observed phenomenon. These findings do not only highlight the importance of signalling pathway crosstalk but they may also suggest a new avenue of combination therapy for some colorectal cancers.

## Background

The Notch signalling pathway, already discovered in 1919 by Thomas H. Morgan in the fruit fly *Drosophila melanogaster*, plays numerous roles in organismal development and tissue homeostasis as well as in different cancers [1-5]. For the activation of Notch signalling, a number of proteolytic processing events are required, most notably the final cleavage of Notch1 by a multi-protein complex termed γ-secretase. This releases a defined fragment (Val1744-NICD) of the membrane bound Notch protein into the cytoplasm, from where it translocates into the nucleus and subsequently mediates the transcription of specific target genes by releasing the repressor activity of CSL (CBF-1/Suppressor of Hairless [Su(H)]/LAG-1; [6]). Recent reports have also documented the existence of additional, 'non-canonical' Notch signalling pathways [7-10].

It has been suggested that inhibition of Notch signalling, for example by γ-secretase inhibition, may be a treatment option for different types of cancers, including colorectal adenocarcinomas (CRC) [7,11-14]. Notch inhibition in normal colon epithelium induces premature differentiation of proliferating cells and treatment of APC^min ^mice, a mouse model of intestinal adenomas, with the potent γ-secretase inhibitor (GSI) dibenzazepine (DBZ) reduces adenomas [5]. However, it was not clear how important Notch signalling is for malignant CRC.

In the current study it is shown that treatment of CRC cells with γ-secretase inhibitors (GSI), which leads to inhibition of Notch signalling, is not sufficient to induce pronounced inhibitory effects on CRC cell proliferation or survival, but results in activation of the MAP kinases Erk1/2. On the other hand, combination of GSI with platinum compounds induced cell death in a substantial subset of CRC cell lines. Inhibition of Erk1/2 can abrogate this combination effect.

## Methods

### Compounds

The GSI compounds DAPT (N- [N-(3,5-difluorophenylacetyl-L-alanyl)]-S-phenylglycine t-butylester; γ-secretase inhibitor IX; 565770) and DBZ [15] ((S, S)-2- [2-(3,5-difluorophenyl)acetylamino]-N-(5-methyl-6-oxo-6,7-dihydro-5H-dibenzo [b, d]azepin-7-yl)propionamide; dibenzazepine; γ-secretase inhibitor XX; 565789) were purchased from Calbiochem (Darmstadt, Germany). The GSI compound L-685,458 (1-benzyl-4-(1-(1-carbamoyl-2-phenylethylcarbamoyl-3-methylbutylcarbamoyl)-2-hydroxy-5-phenylpentyl)carbamic acid t-butylester; L1790) was from Sigma-Aldrich (Poole, Dorset, UK). Three platinum compounds cisplatin (232120; Calbiochem), carboplatin (C2538; Sigma-Aldrich) and oxaliplatin (Eloxatin 5 mg/ml, 248459; Sanofi Aventis, Frankfurt, Germany) were used in this study. The Mek1/2 inhibitor UO126 was from Cell Signaling Technology/NEB (9903; Danvers, MA, USA)

### Antibodies

Polyclonal anti-Notch1 (sc-6014-R) was from Santa Cruz Biotechnology (Santa Cruz, CA, USA), anti-Notch1 mAb (N6786) and anti-actin (A3853) from Sigma-Aldrich. Anti-phospho-Erk1/2 (9101), anti-phospho-Akt (4051), anti-Val1744-NICD (2421) and anti-cleaved PARP (9546) was from Cell Signaling Technology. Anti-Bcl2 (B46620) was from Transduction Laboratories (Lexington, KY, USA). Peroxidase-conjugated anti-mouse (715-036-151) or anti-rabbit IgG (711-036-152) antibodies were from Jackson ImmunoResearch Laboratories (West Grove, PA, USA). Anti-Hes1 was a gift from Dr. Tatsuo Sudo, Toray Industries, Kamakura, Japan.

### Cell lines, cell culture and lysis

The 64 human CRC cell lines used in this study are derived from 63 different patients.

LS 174T and LS 180 originate from the same patient. A full list of the cell lines with a description of their origins is provided in the Additional file 1. For a further characterisation of the 64 cell lines see also Emaduddin et al. [16]. The OXCO-1 and OXCO-3 lines were a gift from Khoon Lin Ling and Vincenzo Cerundolo (WIMM, Oxford).

Cells were grown in Iscove's Modified Dulbecco's Medium (IMDM) supplemented with 100 units/ml penicillin, 100 μg/ml streptomycin and 10% (v/v) FBS at 37°C in humidified atmosphere with 10% CO_2_. Prior to lysis, cells were cultured for 48 h in an excess of medium unless indicated otherwise.

For total cell lysis, cells were washed three times with chilled PBS, lysed in a RIPA buffer (20 mM TrisHCl pH 7.5, 100 mM NaCl, 1 mM EDTA, 1% Triton X-100, 0.5% deoxycholic acid, 0.1% SDS) supplemented with 2× Complete™ protease inhibitor mix (11697498001; Roche Diagnostics, Mannheim, Germany) and phosphatase inhibitor cocktails 1 and 2 (P2850 and P5726; Sigma-Aldrich) and scraped from the culture dishes. Lysates were transferred to microfuge tubes, incubated at 4°C for 30 min on a nutator and then clarified by centrifugation at 22,000 × g for 30 min at 4°C. Protein concentrations were determined by the Bradford method.

To obtain SDS lysates cells were washed three times with PBS at room temperature, residual wash buffer was then removed and cells were scraped with boiling SDS-PAGE sample buffer, followed by 5 min boiling in a hot block. After cooling to room temperature, lysates were sonicated to fragment high molecular weight DNA.

### SDS-PAGE, immunoblotting and immunoprecipitations

Lysates were size-separated by SDS-PAGE and transferred to PVDF membrane (Hybond-P; GE Healthcare, Little Chalfont, Buckinghamshire, UK). Membranes were blocked with 5% skimmed dry milk or 5% BSA dissolved in TBST (20 mM TrisHCl pH 7.5, 100 mM NaCl, 0.1% Tween 20) as suggested by the antibody manufacturer and incubated with primary antibody overnight at 4°C on a nutator. Membranes were then washed 3–4 times for 10–15 min with TBST and incubated for 1 h at room temperature with peroxidase-conjugated anti-mouse or anti-rabbit IgG secondary antibodies. The signals were visualized using ECL detection reagent (32106; Pierce/ThermoScientific, Rockford, IL, USA). Immunoprecipitations were carried out with 1 mg of total cell RIPA lysate using polyclonal anti-NICD. Immunoprecipitates were subjected to immunoblotting analysis with the anti-NICD mAb.

### Cell fractionation

Cells were washed twice with chilled PBS and once with chilled hypotonic lysis buffer (HLB; 10 mM TrisHCl pH 7.5, 10 mM KCl, 1 mM EDTA, 1 mM EGTA, 2 mM MgCl_2_, 1 mM DTT, and protease and phosphatase inhibitors as detailed above). Cells were then scraped with 500 μl HLB, transferrred into a pre-chilled dounce homogeniser and incubated on ice for 15 min. The swollen cells were dounce homogenised with 30 strokes of a tight fitting pestle and the homogenate centrifuged at 1000 × g for 15 min at 4°C. The pellet (P1; contains nuclei) was lysed in RIPA buffer with inhibitors, cleared by centrifugation at 10 000 × g for 30 min at 4°C and the supernatants analysed by immunoblotting (Additional file 2). The supernatant (S1) that remained from the first centrifugation step was further centrifuged at 100 000 × g for 30 min and the supernatant representing the soluble protein fraction (S100) and the pellet (P100) were collected. P100 was lysed in RIPA buffer with inhibitors.

### Cell treatment with compounds

To block γ-secretase, cells were initially treated for 48 h with three different γ-secretase inhibitors, L-685,458, DAPT and DBZ, at concentrations of 5 μM, 10 μM and 300 nM, respectively. Subsequently, to determine whether prolonged inhibition of γ-secretase leads to any visible effects on cell phenotype, treatment was performed for more than a week with daily changes of medium containing inhibitor.

For combination treatment of cells with γ-secretase inhibitor and platinum compounds, DBZ was used for 48 h at 300 nM concentration combined with 1, 3 or 10 μM of cisplatin, oxaliplatin or carboplatin. Cisplatin and carboplatin were always freshly dissolved in DMSO as they are only moderately stable in solution. To block the Mek/Erk pathway, cells were pre-treated with 30 μM of the Mek inhibitor UO126 for 1 h, before further addition of 300 nM DBZ and 10 μM cisplatin for 48 h. Effects on cell growth, survival or morphology were initially analysed by light microscopy (Nikon Eclipse TS100 microscope) and observed changes documented by digital imaging. To analyse changes in cell mass upon drug treatment, cells were fixed and stained with crystal violet solution (0.1% crystal violet in 50% methanol) for 20 min, then washed extensively with water and air-dried. Protein-bound dye was then extracted with 10% acetic acid and the OD of this solution measured at 570 nm.

## Results

### Size heterogeneity of Notch fragments in CRC cells

**Figure 1:**
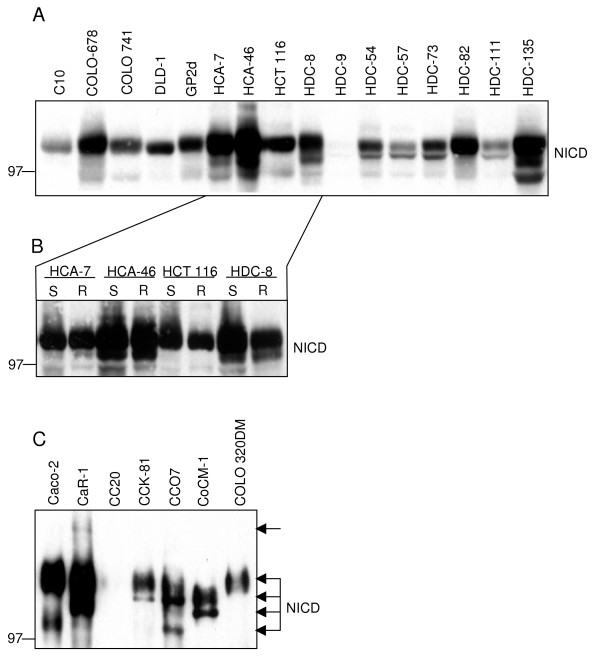
**Expression level and size heterogeneity of Notch fragments detected in CRC cells**. A Western blot detection of Notch fragments in total cell RIPA lysates. 100 μg of total cell protein extracts were subjected to SDS-PAGE and immunoblotting to determine the abundance and sizes of C-terminal fragments of Notch1 (NICD). Results from 16 CRC lines are shown. The heterogeneity in the level of NICD expression and the presence of several NICD fragments of variable length is notable. B Comparison of NICD bands obtained with total cell RIPA lysates (R) and lysates obtained by harvesting cells with boiling SDS-PAGE sample buffer (S). 20 CRC lines were analysed in total. Results from 4 CRC lines are shown as an example. Both protein extraction methods result in very similar NICD band patterns. C Size heterogeneity of immunoprecipitated NICD fragments. NICD fragments were precipitated with a polyclonal antibody (sc-6014-R) directed against the C-terminus of Notch1 and, after extended SDS-PAGE on a large 7% gel, immunoblotted with a mouse mAb (N6786) directed against an epitope in the cdc10-NCR region of Notch1.

To gain insight into potential functions of Notch signalling in CRC cells, initially a panel of 64 CRC cell lines (further details in Additional file 1) was analysed with an antibody raised against the C-terminus of Notch1 for the presence of a Notch fragment corresponding in size to the Notch1 intracellular domain (NICD), which is generated by γ-secretase cleavage of Notch. With this antibody, 63 of 64 CRC lines showed one or more bands corresponding approximately to the expected size. As an example, the results from 16 CRC lines are shown in Figure 1A. The only exception found was the CRC line HDC-9, which was also examined by subcellular fractionation, but no NICD was detected (data not shown).

The other CRC lines differ in their level of NICD expression. In addition, some size heterogeneity of the detected Notch fragments was obvious (Figure 1A). Since the NICD is derived through proteolytic processing, it was important to ensure that signals obtained were not artificially introduced during the experimental procedure as a result of incompletely inhibited proteases. To this end, protein extracts were generated by lysing CRC lines with boiling SDS-PAGE sample buffer (S) and comparing these to lysates obtained with a RIPA-type buffer (R) that contained high concentrations of protease inhibitors. Both types of extracts showed very similar patterns of NICD bands (see Figure 1B for results from 4 of 20 CRC lines tested in total), indicating to us that insufficient protease inhibition does not explain the observed NICD size heterogeneity.

To exclude non-specific signals resulting from antibody cross-reactivity, the NICD was immunoprecipitated using a polyclonal NICD antibody and analysed by immunoblotting with an anti-NICD mAb after protein separation by an extended SDS-PAGE run. As shown in Figure 1C, this confirmed the considerable size heterogeneity of NICD fragments detectable in the CRC lines, possibly a consequence of deregulated proteases in these carcinoma cells.

### The γ-secretase-generated Notch fragment Val1744-NICD is detectable in a subset of CRC cells

**Figure 2:**
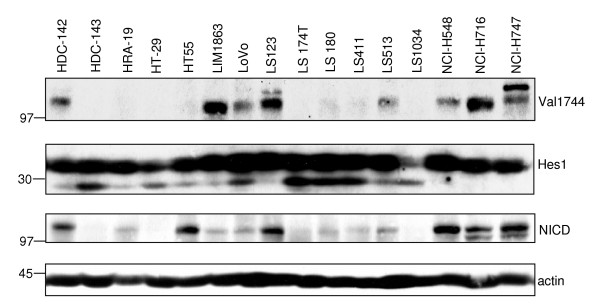
**Variable expression of γ-secretase-cleaved, active Notch1 (Val 1744-NICD) in CRC cells**. 100 μg of total cell RIPA lysates were analysed by immunoblotting using anti-Val1744-NICD antibody. Data from 16 cell lines are shown as an example (upper panel). Lysates were also analysed with anti-Hes1 to determine the expression of this well known Notch1 primary target (second panel from top). A short exposure of a western blot with an antibody directed against the C-terminus of Notch1 reveals that the Val1744-NICD fragment is not always the most prominent fragment present in CRC cells (third panel from top; see for example HT55 cells). Actin was analysed as a loading control (bottom panel).

As some of the NICD fragments detected in CRC may not be functional, the presence of γ-secretase-cleaved, active Val1744-NICD fragments was investigated. Western blotting with a Val1744-NICD fragment-specific antibody showed that approximately half of the CRC lines tested have detectable levels of Val1744-NICD in total cell extracts (see Figure 2, upper panel for examples). However, some of the CRC cell lines that appear to be negative in this experiment still show positive signals for Val1744-NICD after subcellular fractionation in nuclear extracts (see Additional file 2 for details of cell lines analysed). Strikingly, expression of one of the primary Notch target genes, Hes1, does not correlate with the abundance of the Val1744-NICD fragment, suggesting that Notch pathway activity may only fully drive Hes1 expression in some CRC lines and that other pathways could contribute to Hes1 expression regulation in certain CRC cells (Figure 2, second panel from top). On the other hand, very low amounts of Val1744-NICD may be sufficient to drive Hes1 expression in CRC lines. A direct comparison of the obtained Val1744-NICD signals with a short exposure of a blot using the same cell lysates but probed with the antibody raised against the C-terminal region of Notch indicates an imperfect correlation of both Notch-directed antibodies (see for example HT55 cells), again highlighting the importance of determining the presence of Val1744-NICD, which is able to translocate to the nucleus and to induce signalling. Notably, even with the fragment-specific Val1744-NICD antibody more than one protein band is detected in some CRC cell lines (for example in LS123 and NCI-H747). Whether these bands are, for example, due to differential protein modifications remains to be determined. Taken together, these results indicate a great degree of heterogeneity in the Notch fragments present in different CRC cells.

### γ-secretase inhibitors do not elicit striking effects on CRC cell line growth or survival

A primary aim of this study was to determine if Notch signalling is essential for CRC cells. Therefore, in a next step, the potential roles of Notch signalling in CRC cells were investigated in 12 cell lines by inhibiting γ-secretase (Table 1). Nine of these lines detectably expressed Val1744-NICD, albeit in three lines the γ-secretase specific fragment was only detected upon cell fractionation (HCA-7, HCA-46, LoVo; see also Additional file 2). Three CRC lines did not express detectable levels of Val1744-NICD (HDC-9, HDC-57, HDC-73), even after subcellular fractionation.

**Table 1:** Effects of γ-secretase inhibitors and cisplatin on a panel of CRC cell lines

**CRC line**	**Val1744-NICD**	**DAPT **10 μM	**L-685,458 **5 μM	**DBZ **300 nM	**Cisplatin **cell death (20 μM,48 h)	**CP+DBZ **combi effect CD (48 h)
C80	+	-	-	-	+	+
Caco-2	+	-	-	-	+	+
CCK-81	+	-	CD	-	(+)	(+)
CC07	+	-	-	-	+	+
HCA-7	+ (N)	-	-	-	+	++(24 h)
HCA-46	+ (N)	-	-	-	(+)	+
HDC-9	-	-	-	-	+	-
HDC-57	-	-	-	-	-	-
HDC-73	-	-	-	-	+	-
LoVo	+ (N)	-	-	-	+	(+)
LS123	+	-	-	-	+	-
VACO 4A	+	-	CD	-	-	-

CD = cell death visible by light microscopy

CP = cisplatin

CP+DBZ = combination treatment with 10 μM CP and 300 nM DBZ

DAPT = N- [N-(3,5-Difluorophenacetyl-L-alanyl)]-S-phenylglycine t-butyl ester

DBZ = (S, S)-2- [2-(3,5-Difluorophenyl)acetylamino]-N-(5-methyl-6-oxo-6,7-dihydro-5H-dibenzo [b, d]azepin-7-yl)propionamide

(N) = Val1744-NICD detected in nuclear fraction after cell fractionation

NICD = Notch1 intracellular domain

- (minus symbol) = no visible effect on proliferation, cell death or cell morphology

To recognise potential inhibitor off-target effects, three well characterised and structurally distinct GSI, namely DAPT, L-685,458 and DBZ, were directly compared. These were applied in concentrations typically used in the literature (see Table 1 for details) and proven to affect Hes1 expression within CRC cells in initial experiments (data not shown). Cells were treated for 48 h and the detection of prominent effects on cell proliferation, cell survival or cell morphology attempted by light microscopy and cell counting. Surprisingly, inhibition of Notch signalling did not lead to substantial effects on CRC cell growth, morphology or survival with DAPT and DBZ (data not shown). In the case of the L-685,458 inhibitor compound, a moderate degree of cell death was observed in 2 of the 12 CRC lines tested (CCK-81, VACO 4A). However, as all compounds are known to elicit γ-secretase inhibition and DBZ is by far the most potent compound of the three inhibitors tested, the two cell deaths seen upon application of L-685,458 are very likely non-specific, off-target effects. Light microscopic analyses of CRC cells were subsequently continued for over a week, with daily addition of new medium and inhibitor, but without any apparent effect on cell viability, growth or morphology. Some cell lines were also analysed by soft agar colony formation assay over a period of three weeks, again with no obvious effect of GSI application (data not shown).

### γ-secretase inhibition suppresses Hes1 expression and induces Erk activation

The molecular effects of γ-secretase inhibition were then studied in more detail using the Val1744-NICD-positive cell line CCK-81 and comparing DBZ, which is active in nanomolar concentrations and preferentially affects colonic epithelial cells in vivo [15] and the somewhat less potent L-685,458 inhibitor over a time course of 48 h. DAPT appeared to be the least potent of the three inhibitors in first experiments (data not shown) and was not used further.

**Figure 3:**
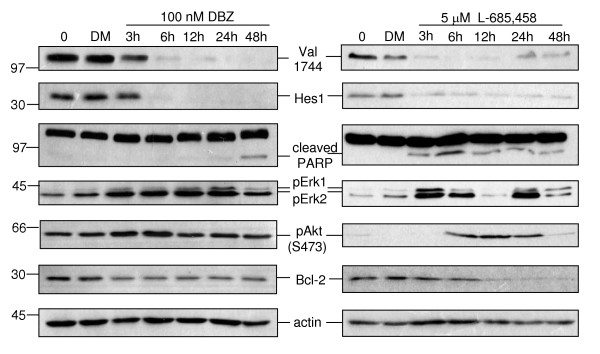
**Molecular effects of γ-secretase inhibition on signalling proteins involved in regulating cell growth or death**. CCK-81 cells were either left untreated (0), treated with DMSO as a control (DM) or incubated with the γ-secretase inhibitors as indicated. Cells were lysed in RIPA buffer and 50 μg of total protein was subjected to immunoblotting analysis as specified. Both inhibitors result in the down-regulation of Val1744-NICD, which is paralleled by a loss of Hes1 expression, an effect that is evident already after 3 to 6 h. Cleaved PARP, an indicator of cell death, was also analysed and more prominent with L-685,458, which induces cell killing in CCK-81 by an unknown mechanism (Table 1). With both GSI, an increase in pErk (pT202pY204) and pAkt (pS473) is evident. In addition, a downregulation of anti-apoptotic Bcl-2 protein was detectable, to a moderate degree by DBZ and more pronounced with L-685458. Actin was analysed as a loading control.

As expected, treatment with either compound substantially reduced the abundance of the Val1744-NICD fragment within a few hours, albeit the effect with DBZ appears to be more pronounced and persistent (Figure 3). However, only a small effect was detected on the cleavage of the caspase substrate poly (ADP-ribose) polymerase (PARP; [17,18]), an indicator of cell death, with 48 h of DBZ treatment, while L-685,458 induced a more rapid response. Erk, a central player in the mitogenic pathway [19,20], and Akt, a well-known cell survival regulator [21], were phosphorylated on key regulatory epitopes (Erk pT202/pY204; Akt pS473) upon treatment of CCK-81 cells with either GSI. Bcl-2, a widely studied, anti-apoptotic protein [22] was moderately reduced in both cases (Figure 3). Erk phosphorylation on the crucial, activity-regulating epitope T202/Y204 was also found repeatedly with other CRC lines analysed, although the kinetics were variable (data for HCA-7 cells shown in Additional file 3). These results prove that, although GSI treatment of CRC cells alone is not sufficient to induce major changes in cell growth or survival, GSI still affect multiple proteins involved in the regulation of these biological functions.

A review of the literature subsequently indicated that some of the molecular effects elicited by GSI in CRC cells could potentially modify the efficacy of existing anti-cancer drugs. For example, it has been reported that the chemotherapeutic drugs like cisplatin and carboplatin depend on Erk activity for their pro-apoptotic effects, since inhibition of Mek/Erk signalling prevented cell death [23-28]. On the other hand, several reports support a different role of Erk in certain types of cancer, associating its activity with enhanced cancer cell survival [29-32].

To determine if GSI can modulate the activity of platinum compounds in CRC cells, DBZ was combined with cisplatin, carboplatin or oxaliplatin in further analyses. All of these compounds are currently in use in the therapy of advanced CRC, but unfortunately none of them is potent enough to cure a substantial number of patients, thus clearly highlighting the urgent need for substantially improved therapies for this frequent cancer type.

### Induction of cell death by combination of GSI and platinum compounds in CRC cells

**Figure 4:**
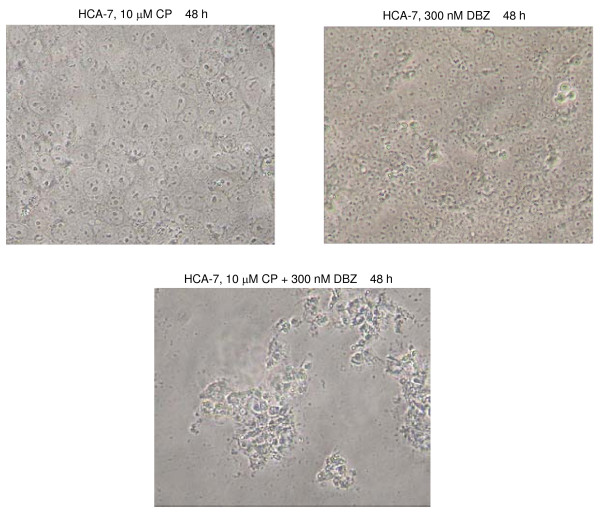
**Combination of Cisplatin with DBZ, a potent GSI, elicits a striking induction of cell death**. Cells were treated as indicated and digital images recorded (20× objective). 10 μM Cisplatin (CP) alone leads to a moderate degree of cell death. Note that less cells are present in cultures treated with cisplatin (upper left panel) compared to untreated or DBZ treated cultures (upper right panel). The cells appear flatter and the nuclei larger. A section of the plate was chosen where only few dead cells obstruct the view onto the remaining cells (see Figure 5 for a lower magnification view of CP treated cells). The combination of cisplatin with DBZ leads to massive cell death.

Different CRC cell lines were first treated with increasing concentrations of cisplatin to establish at what doses cisplatin substantially affects cell survival. While 3 μM cisplatin for 48 h showed typically little effect, cell death was observed with 10 μM cisplatin in many CRC lines, so this dose was used for further drug combination studies. Results from HCA-7 cells are shown here as an example. As readily shown in earlier experiments, application of 300 nM DBZ had no detectable effect on cell survival, but combining 10 μM cisplatin with 300 nM DBZ led to massive cell death (Figure 4). This combination treatment was applied to a total of 20 CRC lines, to determine how frequently an effect can be detected. The results are summarised in Tables 1 and 2. 3 of the 20 CRC lines (HDC-57, COLO 320DM, VACO 4A) appeared to be resistant to cisplatin and no effect of combination treatment with cisplatin and DBZ was seen in these lines. Of the remaining 17 cisplatin-sensitive cell lines, 10 showed at least some degree of increased cell death (Tables 1 and 2), indicating that a major subset of CRC lines is sensitive against the combination of GSI and cisplatin. The effects of inhibitor treatments were also quantified by crystal violet staining of cells. Results are shown in Additional file 4.

**Table 2:** Effects of DBZ and cisplatin on 8 additional CRC cell lines

**CRC line**	**Val1744-NICD**	**DBZ **300 nM	**Cisplatin **cell death (20 μM,48 h)	**CP+DBZ **combi effect CD (48 h)
COLO 320DM	+ (N)	-	-	-
COLO 741	+ (N)	-	+	+
DLD-1	-	-	(+)	-
HCT 116	+ (N)	-	+	+
RCM-1	+	-	+	(+) (72 h)
RKO	+	-	+	(+) (72 h)
SNU-C2B	+	-	(+)	-
SW620	+	-	+	-

For details on abbreviations see footnote in Table 1

**Figure 5:**
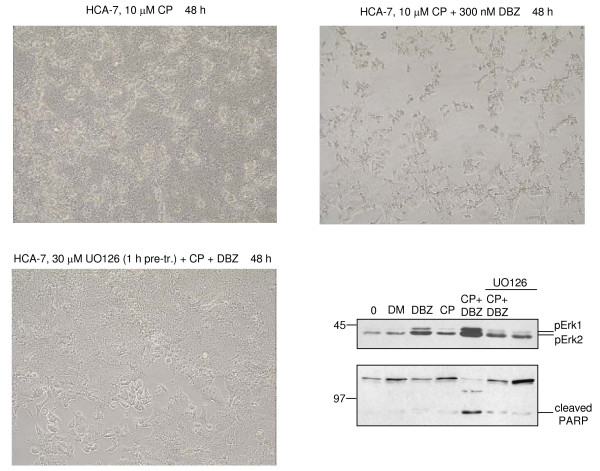
**Inhibition of Erk activation suppresses cell death induced by combining cisplatin and GSI**. Cells were treated as in Figure 4, or additionally pre-incubated for 1 h with the Mek inhibitor UO126 (30 μM) where indicated and digital images recorded (10× objective). Cells were harvested after 24 h and equal amounts of RIPA lysates (50 μg) analysed for pErk and cleaved PARP as before. UO126 blocks the induction of pErk by DBZ and cisplatin, reduces the level of cleaved PARP and suppresses the cell death induced by the combination of these drugs.

All 4 CRC lines that lack detectable Val17744-NICD expression (DLD-1, HDC-9, HDC-57, HDC-73) did not show a cell killing effect upon drug combination, a finding compatible with the hypothesis that inhibition of an active Notch signalling pathway is required for the cell killing effect of DBZ applied together with cisplatin. If this would be correct, introduction of an exogenous Val1744-NICD fragment, which should be unaffected by GSI, into CRC cells, would abolish the combination effect of DBZ and cisplatin treatment obtained with the parental cells. Since transient transfection of CRC cells was only successful for a small percentage of the total CRC cell population in all CRC lines studied, we tried to further test this hypothesis by attempting permanent expression of the Val1744-NICD fragment, but failed so far to obtain clones that stably expressed this Notch fragment. Therefore, we are currently unable to formally exclude that a γ-secretase target other than Notch is linked to the observed drug combination-induced cell killing.

In addition to cisplatin, other platinum derivatives, in particular carboplatin and oxaliplatin are widely used in treating cancer patients. For example, a combination therapy of oxaliplatin with other chemotherapeutic drugs (5-fluorouracil, leucovorin) is now commonly used for treatment of advanced CRC [33]. None of these regimens are, however, even close to being curative for the majority of patients, leaving much room for improved drug combinations.

To detect a potential functional interplay of carboplatin or oxaliplatin with GSI, 5 CRC lines were tested for the effects of combination treatment with 300 nM DBZ and these platinum compounds (10 μM). In HCT 116, HCA-7 and HCA-46 cells drug combination effects were observed. By contrast, the Caco-2 and CC07 cell lines, despite being well responsive to the combination of DBZ and cisplatin, showed no effect with the other two platinum compounds (data not shown). These results were somewhat unexpected, since cisplatin and carboplatin are considered to be quite similar to each other with respect to their mechanism of action and toxicity profile, while oxaliplatin differs considerably with respect to these parameters [34,35]. Clearly, more detailed studies are needed to gain better insight into the differential effects of combining GSI with different platinum compounds.

### Inhibition of Erk activity suppresses cell killing induced by combining of DBZ with cisplatin

The observed Erk activation in CRC cells by GSI could be a bystander effect that is not functionally linked to the cell killing effect observed upon combination of GSI and platinum compounds. In that case, suppression of Erk activity may not quench the observed cell death induced by treatment of cells with cisplatin and DBZ. However, preincubation of HCA-7 cells with the Mek inhibitor UO126, which leads to a reduction of active (pT202pY204) Erk, prior to application of DBZ and cisplatin, clearly reduced the number of killed cells (Figure 5). A reduced cleavage of PARP was also evident when cells were pre-treated with UO126 before the addition of DBZ and cisplatin. This suggests that Mek-Erk signalling plays a functional role in mediating CRC cell killing by combination of GSI and platinum drugs.

## Discussion

Until now, most patients with solid tumors that survive their disease are cured through surgery, often in combination with radiation and/or chemotherapy. Cure rates are especially high for patients with early stage disease. Advanced tumors are in many cases at best delayed in their progression through the use of chemotherapy and/or molecularly targeted drugs. A range of novel molecularly targeted drugs, for example acting against the EGF and IGF receptor families or other tyrosine kinase receptors, PI3 kinase (p110), Akt, mTor, the Wnt pathway, c-Met, Src, CDKs or Aurora kinase are currently in pre-clinical and clinical development [36-38]. However, like most of their predecessors, many of these drug candidates are unfortunately likely to fail, due to a lack of bioactivity and/or dose-limiting adverse effects. Increasing the potency of anti-tumor drugs while limiting their general toxicity therefore remains a very important goal for cancer research.

Platinum compounds are widely used tools in the arsenal of oncologists and currently applied in approximately half of all tumor therapies worldwide. Although cisplatin is one of the few anticancer agents with real curative potential, leading to cure rates beyond 90% in testicular germ cell cancer, its use in CRC has only been moderately successful so far, primarily due to its dose-limiting toxicity. Reducing the overall toxicity of platinum compounds while maintaining or increasing their potency against tumor cells is no easy task. Tumor-specific activation of platinum compounds, although an attractive hypothetical possibility and an active area of research [39], clearly still has a long way to go before it will potentially become a part of the clinical therapy repertoire.

An alternative route to a better usage of existing and newly introduced anti-cancer compounds might be their rational combination with other drugs, based on the individual, patient-specific effects they elicit on the molecular signalling machinery in cancer cells. Again, this is no easy task, but numerous tools and a wealth of molecular knowledge about signalling pathways have been gathered by researchers over the last decades.

The data presented here suggest to us that inhibition of γ-secretase, which abrogates signals from the Notch pathway, could possibly potentiate the in vivo bioactivity of standard chemotherapeutic drugs used in the therapy of colorectal carcinomas and potentially some other cancers. It seems likely to us that the observed cell killing activity elicited by GSI in combination with platinum compounds is not due to a simple overall enhancement of toxicity through drug combination, but that it is cell type-specific instead. Previous studies with the highly potent inhibitor compound DBZ in healthy mice have shown a preferential effect of DBZ on colonic epithelial cells [15]. The DBZ resistance of some colorectal cancer cells that are sensitive to cisplatin would also seem to argue against a general cell toxicity effect and for a more specific combination effect limited to a molecular 'subtype' of CRC. Combining GSI and platinum compounds may therefore create a novel therapeutic window for the treatment of some colorectal cancers.

Whilst there are insufficient data until now to postulate a synergistic effect of DBZ and cisplatin, this intriguing possibility warrants further investigation. Furthermore, despite our encouraging findings with cultured cells, future studies in animal models as well as additional analyses of other platinum compounds and other anti-cancer drugs are clearly needed to decide which drug combinations should be taken forward into clinical testing.

Importantly, this may not be the same combination of drugs for different molecular subtypes of CRCs. At present, it is often impossible to estimate how an individual patient's tumor will respond to a certain therapy. One way to overcome this limitation in the future could be to test primary cancer cells obtained from biopsies, surgery or potentially even tumor cells isolated from patient blood [40] for responses to GSI and platinum compounds. The GSI inhibitor MK-0752 has already shown some activity in T-cell ALL, which frequently harbor mutations in Notch. GSI inhibitors are also currently being tested in breast, CNS and other cancers [41] (further info at ). This provides valuable information on their toxicity, pharmacokinetic and pharmacodynamic properties. Nevertheless, the molecular effects on signalling pathways induced by GSI are only partially known and how Erk activation is induced in CRC cells remains unclear.

In this study, inhibition of Erk was achieved by using the well-characterised Mek inhibitor UO126. Direct selective inhibition of Erk with small molecules is currently probably not feasible since Erk inhibitors typically show spill-over effects onto CDKs, that are, like Erks, proline-directed kinases with considerable similarity in their catalytic clefts.

Previously, links between Notch and the Ras-Mek-Erk pathway have been described in different systems. For example, Notch1 has been described as a target of oncogenic Ras in fibroblasts and Notch inhibition suppressed Ras mediated tumorogenicity in mice [42]. By contrast, mouse mammary tumors resulting from activated Notch4 show activated Mek and Akt and a synergistic relationship between Notch and the Ras signalling pathway has been proposed [43]. In small cell lung cancer cells, overexpression of active Notch1 or 2 led to an increase in Erk activation [44]. From these results it is obvious that very different signalling flows exist between Notch and Erks, which depend, at least in part, on the cell type investigated. Additional analyses are required to determine how GSI increase pErk and pAkt in CRC cells.

As of now, it is not certain that Notch1 is the, or even a, key target mediating GSI effects seen in CRC. A number of other γ-secretase substrates are known, including the signalling proteins ErbB4 [45], IGF1R [46], E-Cadherin and DCC [47]. Expression of active Notch1 fragments in several CRC lines by using viral vectors should be able to shed some light onto this open question rather quickly.

## Conclusion

The results presented here once again highlight the molecular diversity of lesions in cancer cells originating from the same tissue and suggest that the combination of GSI with platinum compounds may provide an option to improve treatments for a subset of CRC patients.

## Abbreviations

CRC: colorectal cancer; DMSO: dimethyl sulphoxide; GSI: γ-secretase inhibitor; NICD: Notch intracellular domain; PARP: poly (ADP-ribose) polymerase; TBST: TBS with 0.1% Tween 20.

## Competing interests

The authors declare that they have no competing interests.

## Authors' contributions

TA and SF designed and conducted experiments, analysed data and drafted the manuscript.

## Supplementary Material

Additional file 1 **Origin of 64 CRC cell lines.** sources of purchase or references of publications that describe the generation of the lines. Click here for file

Additional file 2 **Search for Val1744-NICD in subcellular fractions.** Western blots of cell fractions from selected CRC cell lines that did not show Val1744-NICD in total cell lysates. Click here for file

Additional file 3 **Kinetics of Erk and Akt activation upon GSI treatment.** Molecular effects of γ-secretase inhibition on signalling proteins involved in regulating cell growth or death in HCA-7 cells. Click here for file

Additional file 4 **Quantification of DBZ and cisplatin effects on CRC cell growth.** Crystal violet staining of 20 CRC cell lines fixed after treatment with DBZ, cisplatin or a combination of both. Click here for file
