# Supplementary material for: Gamma-secretase inhibition combined with platinum compounds enhances cell death in a large subset of colorectal cancer cells
Source: Cell Commun Signal. 2008 Oct 24;6:8. doi: 10.1186/1478-811X-6-8 (PMC2584637; doi:10.1186/1478-811X-6-8)
Supplement: Additional file 1 — Origin of 64 CRC cell lines. sources of purchase or references of publications that describe the generation of the lines. [file 1478-811X-6-8-S1.doc]

**Additional file 1: Colorectal cancer cell line panel**

| **No.** | Name* | Reference / Commercial Source |
| --- | --- | --- |
| 1. | C10 | (1) |
| 2. | C32 | (1) |
| 3. | C70 | (1) |
| 4. | C75 | (1) |
| 5. | C80 | (1) |
| 6. | C84 | (1) |
| 7. | C99 | (2) |
| 8. | C106 | (2) |
| 9. | C125-PM | (3) |
| 10. | Caco-2 | ATCC, USA (www.atcc.org) |
| 11. | CaR-1 | Health Science Research Resource Bank (HSRRB), Japan (www.jhsf.or.jp) |
| 12. | CC20 | (4) |
| 13. | CCK-81 | HSRRB |
| 14. | CC07 | (1) |
| 15. | CoCM-1 | HSRRB |
| 16. | COLO 320DM | ATCC |
| 17. | COLO-678 | DSMZ, Germany (www.dsmz.de) |
| 18. | COLO 741 | ECACC, UK (www.ecacc.org.uk) |
| 19. | DLD-1 | ATCC |
| 20. | GP2d | (5) |
| 21. | HCA-7 | (6) |
| 22. | HCA-46 | (7) |
| 23. | HCT 116 | ATCC |
| 24. | HDC-8 | (8) |
| 25. | HDC-9 | (8) |
| 26. | HDC-54 | (8) |
| 27. | HDC-57 | (8) |
| 28. | HDC-73 | (8) |
| 29. | HDC-82 | (8) |
| 30. | HDC-111 | (8) |
| 31. | HDC-114 | (8) |
| 32. | HDC-135 | (8) |
| 33. | HDC-142 | (8) |
| 34. | HDC-143 | (8) |
| 35. | HRA-19 | (9) |
| 36. | HT-29 | ATCC |
| 37. | HT55 | (10) |
| 38. | LIM1863 | (11) |
| 39. | LoVo | ATCC |
| 40. | LS123 | ATCC |
| 41. | LS 174T | ATCC |
| 42. | LS 180 | ATCC |
| 43. | LS411 | ATCC |
| 44. | LS513 | ATCC |
| 45. | LS1034 | ATCC |
| 46. | NCI-H548 | ATCC |
| 47. | NCI-H716 | ATCC |
| 48. | NCI-H747 | ATCC |
| 49. | OXCO-1 | Vincenzo Cerundolo and Khoon Lin Ling, WIMM, Oxford, UK (Unpublished) |
| 50. | OXCO-3 | Vincenzo Cerundolo and Khoon Lin Ling, WIMM, Oxford, UK (Unpublished) |
| 51. | PC/JW | (12) |
| 52. | RCM-1 | HSRRB |
| 53. | RKO | ATCC |
| 54. | SK-CO-1 | ATCC |
| 55. | SNU-C2B | ATCC |
| 56. | SW48 | ATCC |
| 57. | SW403 | ATCC |
| 58. | SW620 | ATCC |
| 59. | SW837 | ATCC |
| 60. | SW948 | ATCC |
| 61. | SW1116 | ATCC |
| 62. | SW1417 | ATCC |
| 63. | T84 | ATCC |
| 64. | VACO 4A | (13) |

*Cell line designations according to cited reference or use by commercial source.

**References for Additional file 1**

1. Browning MJ, Krausa P, Rowan A, Bicknell DC, Bodmer JG*, et al.* (1993) Tissue typing the hla-a locus from genomic DNA by sequence-specific pcr: Comparison of hla genotype and surface expression on colorectal tumor cell lines. *Proc Natl Acad Sci U S A* 90:2842-2845.

2. Wheeler JM, Beck NE, Kim HC, Tomlinson IP, Mortensen NJ*, et al.* (1999) Mechanisms of inactivation of mismatch repair genes in human colorectal cancer cell lines: The predominant role of hmlh1. *Proc Natl Acad Sci U S A* 96:10296-10301.

3. Liu Y, Bodmer WF (2006) Analysis of p53 mutations and their expression in 56 colorectal cancer cell lines. *Proc Natl Acad Sci U S A* 103:976-981.

4. Greenhalgh DA, Kinsella AR (1985) C-ha-ras not c-ki-ras activation in three colon tumour cell lines. *Carcinogenesis* 6:1533-1535.

5. Solic N, Collins JE, Richter A, Holt SJ, Campbell I*, et al.* (1995) Two newly established cell lines derived from the same colonic adenocarcinoma exhibit differences in egf-receptor ligand and adhesion molecule expression. *Int J Cancer* 62:48-57.

6. Kirkland SC (1985) Dome formation by a human colonic adenocarcinoma cell line (hca-7). *Cancer Res* 45:3790-3795.

7. Kirkland SC, Bailey IG (1986) Establishment and characterisation of six human colorectal adenocarcinoma cell lines. *Br J Cancer* 53:779-785.

8. Bruderlein S, van der Bosch K, Schlag P, Schwab M (1990) Cytogenetics and DNA amplification in colorectal cancers. *Genes Chromosomes Cancer* 2:63-70.

9. Kirkland SC (1986) Endocrine differentiation by a human rectal adenocarcinoma cell line (hra-19). *Differentiation* 33:148-155.

10. Watkins JF, Sanger C (1977) Properties of a cell line from human adenocarcinoma of the rectum. *Br J Cancer* 35:785-794.

11. Whitehead RH, Jones JK, Gabriel A, Lukies RE (1987) A new colon carcinoma cell line (lim1863) that grows as organoids with spontaneous differentiation into crypt-like structures in vitro. *Cancer Res* 47:2683-2689.

12. Berry RD, Paraskeva C (1988) Expression of carcinoembryonic antigen by adenoma and carcinoma derived epithelial cell lines: Possible marker of tumour progression and modulation of expression by sodium butyrate. *Carcinogenesis* 9:447-450.

13. McBain JA, Weese JL, Meisner LF, Wolberg WH, Willson JK (1984) Establishment and characterization of human colorectal cancer cell lines. *Cancer Res* 44:5813-5821.
