# Supplementary material for: Gamma-secretase inhibition combined with platinum compounds enhances cell death in a large subset of colorectal cancer cells
Source: Cell Commun Signal. 2008 Oct 24;6:8. doi: 10.1186/1478-811X-6-8 (PMC2584637; doi:10.1186/1478-811X-6-8)
Supplement: Additional file 2 — Search for Val1744-NICD in subcellular fractions. Western blots of cell fractions from selected CRC cell lines that did not show Val1744-NICD in total cell lysates. [file 1478-811X-6-8-S2.ppt]

## Slide 1
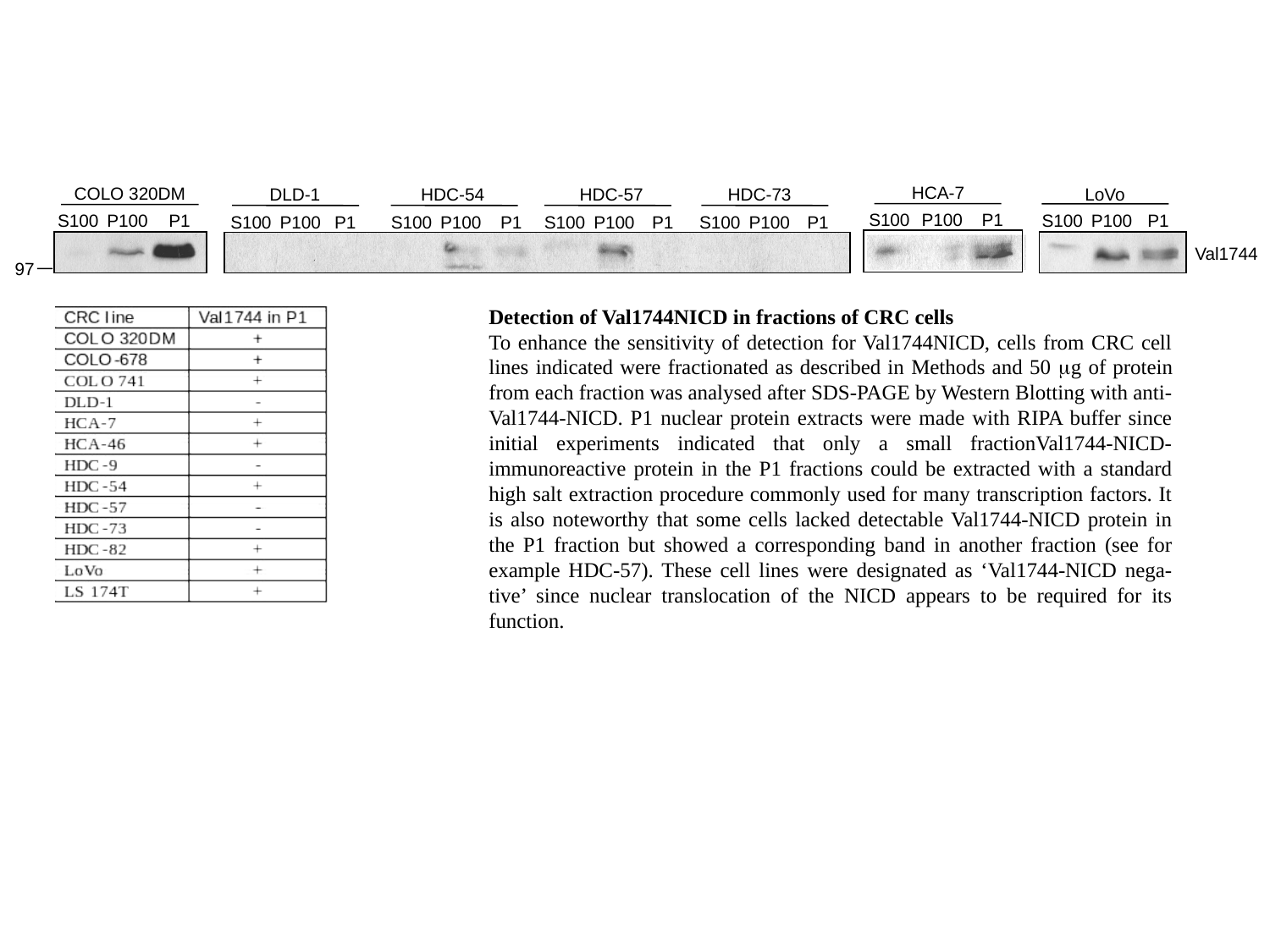

HCA-7
COLO 320DM
DLD-1
HDC-54
HDC-57
HDC-73
LoVo
S100
P100
P1
S100
P100
S100
P100
P1
P1
S100
P100
P1
S100
P100
P1
S100
P100
P1
S100
P100
P1
Val1744
97
Detection of Val1744NICD in fractions of CRC cells
To enhance the sensitivity of detection for Val1744NICD, cells from CRC cell lines indicated were fractionated as described in Methods and 50 μg of protein from each fraction was analysed after SDS-PAGE by Western Blotting with anti-Val1744-NICD. P1 nuclear protein extracts were made with RIPA buffer since initial experiments indicated that only a small fractionVal1744-NICD-immunoreactive protein in the P1 fractions could be extracted with a standard high salt extraction procedure commonly used for many transcription factors. It is also noteworthy that some cells lacked detectable Val1744-NICD protein in the P1 fraction but showed a corresponding band in another fraction (see for example HDC-57). These cell lines were designated as ‘Val1744-NICD nega-tive’ since nuclear translocation of the NICD appears to be required for its function.
