# Supplementary material for: Gamma-secretase inhibition combined with platinum compounds enhances cell death in a large subset of colorectal cancer cells
Source: Cell Commun Signal. 2008 Oct 24;6:8. doi: 10.1186/1478-811X-6-8 (PMC2584637; doi:10.1186/1478-811X-6-8)
Supplement: Additional file 3 — Kinetics of Erk and Akt activation upon GSI treatment. Molecular effects of γ-secretase inhibition on signalling proteins involved in regulating cell growth or death in HCA-7 cells. [file 1478-811X-6-8-S3.ppt]

## Slide 1
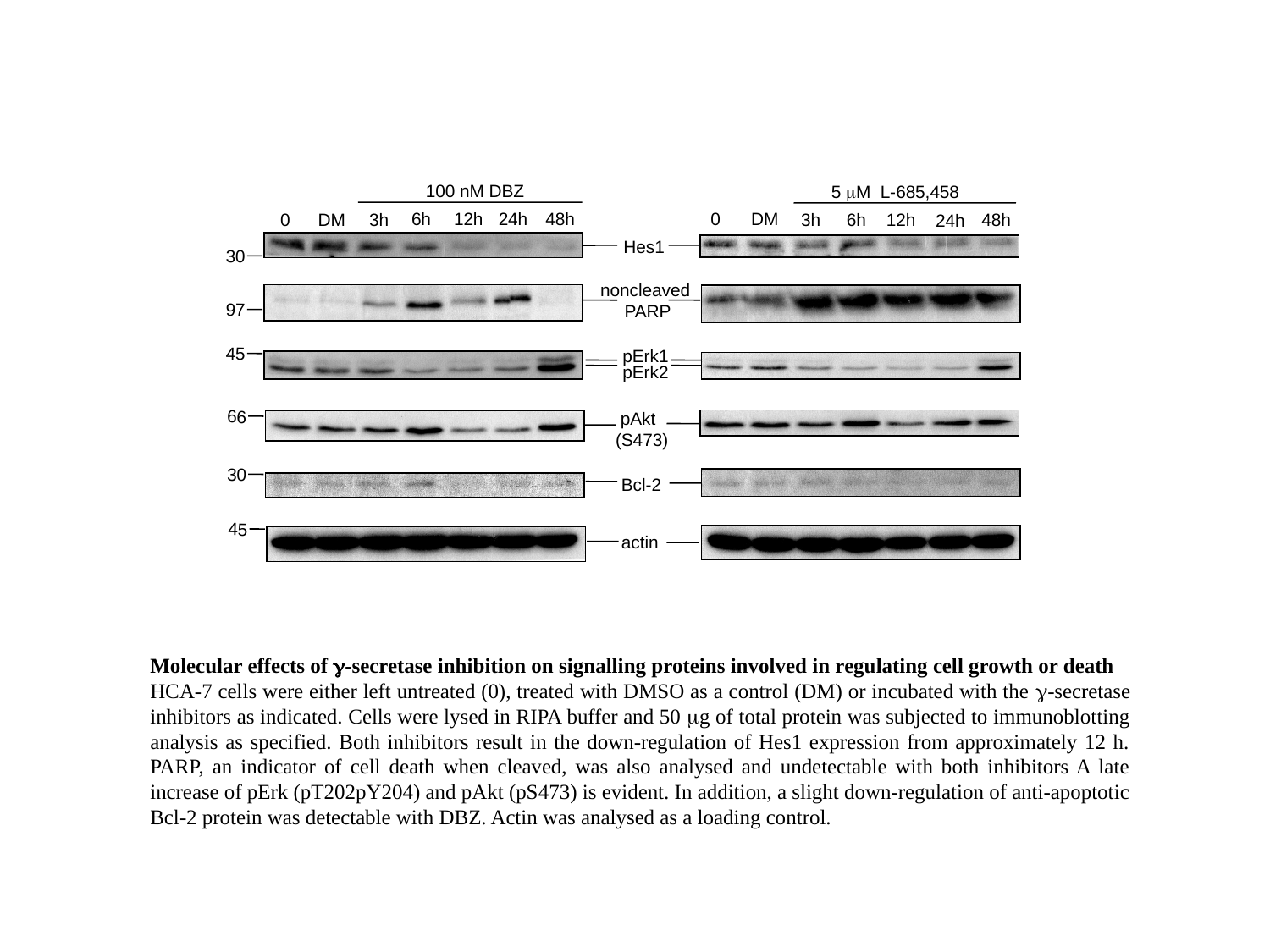

100 nM DBZ
5 μM L-685,458
6h
12h
24h
48h
0
DM
0
DM
3h
3h
6h
12h
48h
24h
 Hes1
30
noncleaved
 PARP
97
45
 pErk1
 pErk2
66
 pAkt
(S473)
30
 Bcl-2
45
 actin
Molecular effects of γ-secretase inhibition on signalling proteins involved in regulating cell growth or death
HCA-7 cells were either left untreated (0), treated with DMSO as a control (DM) or incubated with the γ-secretase inhibitors as indicated. Cells were lysed in RIPA buffer and 50 μg of total protein was subjected to immunoblotting analysis as specified. Both inhibitors result in the down-regulation of Hes1 expression from approximately 12 h. PARP, an indicator of cell death when cleaved, was also analysed and undetectable with both inhibitors A late increase of pErk (pT202pY204) and pAkt (pS473) is evident. In addition, a slight down-regulation of anti-apoptotic Bcl-2 protein was detectable with DBZ. Actin was analysed as a loading control.
